# Concomitant induction of SLIT3 and microRNA-218–2 in macrophages by toll-like receptor 4 activation limits osteoclast commitment

**DOI:** 10.1186/s12964-023-01226-w

**Published:** 2023-08-18

**Authors:** Eun-Young Kim, Ji-Eun Kim, Soo-Hyun Chung, Ji-Eun Park, Dohee Yoon, Hyo-Jin Min, Yoolim Sung, Soo Been Lee, Seong Who Kim, Eun-Ju Chang

**Affiliations:** 1 Department of Biochemistry and Molecular Biology, Asan Medical Center and AMIST, University of Ulsan College of Medicine, 88, Olympic-Ro 43-Gil, Songpa-Gu, Seoul, 05505 Korea; 2 Stem Cell Immunomodulation Research Center, University of Ulsan College of Medicine, Seoul, 05505 Korea

**Keywords:** SLIT3, Macrophages, Toll-like receptor 4 (TLR4), miR-218–2-3p, RANK, Osteoclast commitment

## Abstract

**Background:**

Toll-like receptor 4 (TLR4) conducts a highly regulated inflammatory process by limiting the extent of inflammation to avoid toxicity and tissue damage, even in bone tissues. Thus, it is plausible that strategies for the maintenance of normal bone-immunity to prevent undesirable bone damage by TLR4 activation can exist, but direct evidence is still lacking.

**Methods:**

Osteoclast precursors (OCPs) obtained from *WT* or *Slit3*-deficient mice were differentiated into osteoclast (OC) with macrophage colony-stimulating factor (M-CSF), RANK ligand (RANKL) and lipopolysaccharide (LPS) by determining the number of TRAP-positive multinuclear cells (TRAP^+^ MNCs). To determine the alteration of OCPs population, fluorescence-activated cell sorting (FACS) was conducted in bone marrow cells in mice after LPS injection. The severity of bone loss in LPS injected *WT* or *Slit3*-deficient mice was evaluated by micro-CT analysis.

**Result:**

We demonstrate that TLR4 activation by LPS inhibits OC commitment by inducing the concomitant expression of *miR-218–2-3p* and its host gene, *Slit3*, in mouse OCPs. TLR4 activation by LPS induced SLIT3 and its receptor ROBO1 in BMMs, and this SLIT3-ROBO1 axis hinders RANKL-induced OC differentiation by switching the protein levels of C/EBP-β isoforms. A deficiency of SLIT3 resulted in increased RANKL-induced OC differentiation, and the elevated expression of OC marker genes including *Pu.1*, *Nfatc1*, and *Ctsk*. Notably, *Slit3*-deficient mice showed expanded OCP populations in the bone marrow. We also found that miR-218–2 was concomitantly induced with SLIT3 expression after LPS treatment, and that this miRNA directly suppressed *Tnfrsf11a* (RANK) expression at both gene and protein levels, linking it to a decrease in OC differentiation. An endogenous *miR-218–2* block rescued the expression of RANK and subsequent OC formation in LPS-stimulated OCPs. Aligned with these results, *SLIT3*-deficient mice displayed increased OC formation and reduced bone density after LPS challenge.

**Conclusion:**

Our findings suggest that the TLR4-dependent concomitant induction of *Slit3* and *miR-218–2* targets RANK in OCPs to restrain OC commitment, thereby avoiding an uncoordinated loss of bone through inflammatory processes. These observations provide a mechanistic explanation for the role of TLR4 in controlling the commitment phase of OC differentiation.

Video Abstract

**Supplementary Information:**

The online version contains supplementary material available at 10.1186/s12964-023-01226-w.

## Background

Monocyte-macrophage lineages differentiate into inflammatory macrophages during the immune response to pathogens [1] and serve as precursors of bone-resorbing osteoclasts (OCs), which are responsible for the degradation of the bone matrix, essential for the maintenance of bone homeostasis [2]. c-Fms (CSF1R)^+^RANK^+^ OC precursors (OCPs) are committed pre-fusion OC (pOC) that can terminally differentiate into OCs during OC formation [3, 4]. During the commitment phase of the OC differentiation program, it is a prerequisite that macrophage colony-stimulating factor (M-CSF) binds to its receptor c-Fms to induce the expression of the receptor activator of nuclear factor κB (RANK) required for pOC formation [5, 6]. Hence, the timing of RANK expression in OCPs and its subsequent binding of RANK ligand (RANKL) are critical steps in OC differentiation [5, 6].

Toll-like receptor 4 (TLR4) is one of the best-characterized pattern recognition receptors in macrophages that respond to infection and microbial products, and is closely associated with bone homeostasis and inflammatory bone disease [7–9]. It is important to note that TLR4 activation by lipopolysaccharide (LPS) shows a discrepancy between in vivo and in vitro OC formation. TLR4 activation in vivo simultaneously induces OC-driven bone resorption and inhibits osteoblast (OB) differentiation, resulting in an increase in bone loss [10]. TLR4 increases the survival, fusion, and activation of OCs by regulating NFATc1 expression and activating the Akt, NF-κB, and ERK pathways acts through their actions on RANK-expressing OCPs [11, 12]. This process is orchestrated via other pro-inflammatory cytokines such as IL-1 and TNF-α in inflammatory bone diseases in vivo [7] by stimulating OC formation from macrophages directly [13] and inducing RANKL production in pre-osteoblasts (pOBs) to enhance OC forming potential [14]. During OC differentiation, the TLR4 activation enhances OC differentiation in RANK-expressing OCPs [11]. In contrast, TLR4 activation plays a key role in the fate of monocytes by promoting their conversion into macrophages and blocking OCP formation during the commitment phase [11] through the inhibition of RANK and c-Fms in monocytes/macrophages [11, 15, 16]; however, the underlying molecular mechanisms remain poorly defined.

Secreted SLIT glycoproteins, including SLIT1, -2, and -3, regulate cell-environment interactions via the mediation of roundabout (ROBO) receptors [17]. The SLIT/ROBO pathway is associated with the regulation of cell migration [18, 19], apoptosis [20], proliferation [21], adhesion, and angiogenesis [22], and has a critical role in various cell types such as lung, kidney, liver, breast, and immune cells (*e.g.*, macrophages), indicating a role in many diseases and inflammatory states [23]. SLIT3 is ubiquitously expressed in a wide variety of tissues and plays a role in monocyte migration by activating RhoA [24]. It was previously reported that LPS upregulates SLIT3 expression and thereby mediates the migration of mouse peritoneal macrophages through Rac/Cdc42 activation [25]. It therefore important to define whether upregulated SLIT3 in LPS-stimulated OCPs can affect OC differentiation.

In the present study, we found that TLR4-mediates SLIT3 induction in OCPs, and that this hinders OC differentiation by controlling OC commitment. Moreover, we observed that both LPS-induced SLIT3 and *miR-218–2*, which targets *Tnfrsf11a* (RANK), regulate the decrease in OCP formation. These results provide important insights into the molecular mechanisms by which TLR4 activation controls OC differentiation through a block of OCP formation during the macrophage development commitment phase.

## Materials and methods

### Mice

We purchased 6- or 8-week-old female C57BL/6 J (*WT*) mice and *Tlr4* mutant (*Tlr4*^*LPS−del*^, referred as *Tlr4*^−/−^) mice on a C57BL/6 J background from The Jackson Laboratory (Bar Harbor, ME). SLIT3 KO (*Slit3*^−/−^) mice were generated previously [26]. The Institutional Animal Care and Use Committee of the Asan Institute for Life Sciences (Seoul, Korea) approved all animal experiments.

### Cell culture

OCPs were prepared essentially as described in previous reports with some modifications [27, 28]. Briefly, bone marrow (BM) cells were isolated through flushing from the tibia, treated with red blood cell lysis buffer (Invitrogen; Carlsbad, CA) to eliminate red blood cells, and cultured overnight in α-MEM (HyClone Laboratories; Logan, UT) containing 10% heat-inactivated fetal bovine serum (FBS) (HyClone Laboratories), penicillin (100 U/mL), and streptomycin (100 mg/mL) (HyClone Laboratories). Non-adherent cells were then harvested and cultivated in 10-cm Petri dishes (SPL, Gyeonggi-do, Korea) supplemented with M-CSF (30 ng/mL) (Peprotech, Cranbury, NJ). Adherent cells were harvested after three days and used as OCPs. To stimulate these cells with LPS, they were cultured overnight in 6-well plates (3 × 10^5^ cells /well), and then treated with 10 ng/mL LPS prepared from *E. coli* strain O26:B6 (Sigma-Aldrich, St. Louis, MO). To differentiate OCs in vitro, the prepared OCPs were cultured with 30 ng/mL M-CSF plus 10 ng/mL RANKL (R&D systems, Minneapolis, MN) in the presence or absence of LPS for four days. To analyze the roles of SLIT3 in OC differentiation, the growth medium was supplemented with rSLIT3 for indicated times. To monitor OC differentiation, the cells were fixed, stained using a Leukocyte Acid Phosphatase (TRAP) kit (Sigma-Aldrich) in accordance with the manufacturer’s instruction, and observed under a light microscope. OCs were identified as TRAP^+^ MNCs (nuclei ≥ 3). TRAP^+^ multinucleated giant cells in the 48-well plates were counted using a Nikon ECLIPSE TS100 microscope and photographed using a Nikon DS-U3 camera. TRAP^+^ MNCs were counted by an investigator blinded to the condition of the subjects.

### Quantitative real-time PCR (qPCR)

Total RNA extraction, cDNA synthesis, and qPCR analyses were performed as previously described [29]. The sequences of the oligonucleotide primers used were as follows: 5′-CTAAACCAGACCCTGAACCTGGTGGTAGA-3′ (*Slit3* forward), 5′-AAGGTAGAGGGGGCTGTTGCTGCCCACT-3′ (*Slit3* reverse); 5′-AGGGAAGCCTACGCAGAT-3′ (*Robo1* forward), 5′-TGGACAGTGGGCGATTTTAT-3′ (*Robo1* reverse); 5’-CAGATGTCTTTTCGTCCACAGA-3’ (*Tnfrsf11* forward), 5’-AGACTGGGCAGGTAAGCCT-3’ (*Tnfrsf11* reverse); 5’AATACCTCCCTCTCGATCCTACA-3’ (*Ctsk* forward), 5’-GGTTCTTGACTGGAGTAACGTA-3’ (*Ctsk* reverse); 5’-GATGGAGAAGCTGATGGCTTGG-3’ (*Pu.1* forward), 5’-TTCTTCACCTCGCCTGTCTTGC-3’ (*Pu.1* reverse); 5’-TTCTTCACCTCGCCTGTCTGC-3’ (*Nfatc1* forward), 5’-GGAAGTCAGAAGTGGGTGGA-3’ (*Nfatc1* reverse); 5’-GGGTTGTTGATGTTTTTGGTT-3’ (*C/ebp-β* forward), 5’-GAAACGGAAAAGGTTCTCAAAA-3’ (*C/ebp-β* reverse); 5′-TGGCTTTCCGTGTTCCTAC-3′ (*Gapdh* forward), and 5′-GAGTTGCTGTTGAAGTCGCA-3′ (*Gapdh* reverse). qPCR was performed using a LightCycler 480 SYBR Green I-Step Kit and the LightCycler® 480 Instrument II Real Time PCR System (Roche Applied Science; Mannheim, Germany) in accordance with the manufacturer’s instructions. For the detection of *miR-218–2-3p*, cDNAs prepared by reverse transcription using the miScript HiFlex Buffer from the miScript II RT Kit (Qiagen, Germantown, MD) were subjected to qPCR using an miRNA-specific miScript Primer Assay (forward primer) and the miScript SYBR Green PCR Kit, which contains the miScript Universal Primer (reverse primer) and QuantiTect SYBR Green PCR Master Mix (Qiagen).

### Immunoblotting

Immunoblotting was performed as previously described [30]. Briefly, cell lysates were prepared using RIPA buffer (Thermofisher, Rockford, IL). These preparations were then electrophoresed by SDS-PAGE, and the resolved proteins were transferred to a polyvinylidene difluoride membrane (Bio-Rad, Hercules, CA). Non-specific interactions were blocked using a 5% bovine serum albumin solution in Tris-buffer saline (20 mM Tris/HCl, pH7.6, 150 mM NaCl, and 0.1% Triton X-100) for 1 h. The membranes were then incubated with primary antibodies against C/EBP- β (Abcam, Cambridge, UK), SLIT3 (Adnova, Taipei, Taiwan), NFATc1 (Abcam), and RANK (Thermofisher) overnight at 4 ℃. Subsequently, membranes were incubated with the appropriate secondary antibodies conjugated with horseradish peroxidase and immunoreactivity was detected using an ECL kit (Millipore, Billerica, MA).

### Gene silencing and transfection

The transfection of siRNAs targeting *Slit3* was performed as previously described [31]. The combination of four select siRNA oligonucleotides, i.e., a SMARTpool siRNA targeting *Slit3* (ON-TARGET plus mouse *Slit3*), and negative control siRNA were purchased from Thermo Scientific Dharmacon (Lafayette, CO). BMMs were transfected with these siRNAs using the RNAiMAX transfection reagent (Invitrogen; Carlsbad, CA). The knockdown of *Slit3* in LPS-treated cells was confirmed at both the mRNA and protein levels (Figure S1A, B). An miRNA mimic (*miR-218–2-3p*) and its inhibitor anti-miR (*anti-miR-218–2-3p*) were purchased from Qiagen for miRNA functional assays. The molecules were transiently transfected into OCPs using RNAiMAX.

### Immunofluorescence confocal microscopy

OCPs were grown on glass coverslips and incubated for 6 h with or without LPS (10 ng/mL). These cells were then fixed with 4% paraformaldehyde in phosphate-buffered saline (PBS) for 15 min at room temperature and permeabilized with 0.1% Triton X-100. The cells were blocked with 1% bovine serum albumin in PBS for 1 h and then incubated with anti-SLIT3 antibody (Adnova) or anti-Robo1 antibody (Abcam) overnight at 4 ℃. After washing, the cells were incubated with Alexa Fluor 488 dye-conjugated anti-rabbit IgG antibody (Santa Cruz Biotechnology, Santa Cruz, CA) and counterstained with DAPI (Invitrogen, Grand Island, NY). The cells were then mounted and SLIT3 protein expression was detected using a confocal laser-scanning microscope (LSM 710; Carl Zeiss).

### Enzyme-linked immunosorbent assay (ELISA) for SLIT3

For the detection of SLIT3 by ELISA, OCPs were seeded in 6-well plates and grown to 70% confluence. These cells were then incubated with vehicle or 10 ng/mL LPS for 24 h and additionally incubated for 24 h with LPS. The level of secreted SLIT3 protein in the conditioned medium and in cell lysates was measured using a mouse SLIT3 ELISA kit (Antibody-online Inc., Atlanta, GA) in accordance with the manufacturer’s instructions. Briefly, supernatants or lysates from the cell cultures were incubated in the pre-coated wells for 3 h at room temperature. This was followed by a 1-h incubation with biotinylated anti-mouse SLIT3 detection antibodies diluted to 0.5 μg/mL also at room temperature. Tetramethyl benzidine substrates were added and the OD values were determined at 450 nm. All samples were measured in triplicate wells, and the average absorbance of the blank wells was subtracted from the measured values.

### LPS-induced bone resorption model

An LPS (5 mg/kg) challenge was performed by two intraperitoneal injections of *WT* and *Slit3*^−/−^, at a three-day interval. To analyze OCP populations in the BM, the mice were sacrificed the next day, and BM cells were harvested and analyzed by flow cytometric analysis as described below. To analyze bone resorption activity in vivo, mice were sacrificed four days after the second LPS challenge. The hind limbs were also dissected, fixed in 4% paraformaldehyde (PFA), and examined for bone resorption activity by micro-computed tomography (CT) analysis. To differentiate OCs with BM cells from PBS or LPS injected mice, the isolated BM cells were cultured with M-CSF (30 ng/mL) and RANKL (10 ng/mL) for six days. We conducted TRAP staining as aforementioned. Fixed hind limbs were also used for histological analysis. Briefly, bone tissues were maintained in EDTA decalcification solution for 30 days and underwent hematoxylin and eosin staining or TRAP staining as described previously [32]. TRAP-positive areas were assessed by using ImageJ densitometry software.

### Flow cytometric analysis

To analyze the frequency of OCP subsets, BM cells were collected from the mice, and washed three times with PBS supplemented with 4% FBS. After washing, the cells were treated with an anti-mouse CD16/CD32 antibody (BD, San Jose, CA) for 15 min on ice to block non-specific binding. The cells were then stained with APC-Cy7-conjugated anti-mouse CD45 antibody (Biolegend, San Diego, CA), FITC-conjugated anti-mouse CD117 antibody (Biolegend, San Diego, CA), PE-conjugated anti-mouse CD115 antibody (Biolegend, San Diego, CA), or APC-conjugated anti-CD11b antibody (Biolegend, San Diego, CA) for 30 min on ice. The cells were washed with PBS with 4% FBS, and analyzed using a Canto II (BD Biosciences, San Jose, CA) analyzer and Flowjo software to process the data (Tree Star, Ashland, OR). Monocytes/macrophages obtained from mouse bone marrow were cultured with M-CSF (30 ng/ml) for 3 days and treated with LPS (10 ng/ml) for 2 more days and were collected to analyze the RANK expression. APC-conjugated anti-mouse CD265 (RANK) antibody (Biolegend, San Diego, CA) was used to observe the RANK expression in OCPs.

### Micro-CT analysis

Femoral bones from the experimental mice were evaluated by micro-CT analysis as described previously [33]. Briefly, fixed bones from the hind limbs of the animals were scanned using the Skyscan 1072 system (Skyscan, Kontich, Belgium). After the acquisition of tomographic slices, bone volume analysis was performed and three-dimensional surface-rendered models were produced through the CTAn and CTvol software (Bruker-micro-CT).

### Plasmid constructs

The *Tnfrsf11a* (RANK) 3’-UTR was amplified by PCR from mice-isolated genomic DNA. The *Tnfrsf11a* (RANK) 3’-UTR was amplified by PCR from mouse OCP-isolated genomic DNA. The pMIR-RANK-3’-UTR construct was digested with *HindIII* and *SacI*, and the generated fragment was inserted into the corresponding restriction sites of the pMIR-REPORT miRNA Expression Reporter vector (Applied Biosystems, Carlsbad, CA). Two *miR-218–2-3p* binding sites in the *Tnfrsf11a* 3’-UTR were predicted using the miRanda-mirSVR application system and were located at 3,365–3,370 bp and 3,458–3,467 bp relative to the transcription start site. Mutations were made in the *miR-218–2-3p* binding sites using primers described in Fig. 3G in the *Tnfrsf11a* 3’-UTR using the Muta-DirectTM Site-directed mutagenesis kit (Intron, Seoul, Korea) in accordance with the manufacturer’s protocol.

### Luciferase reporter assay

NIH3T3 cells were transfected for 24 h with either *WT* or mutant pMIR REPORT-*Tnfrsf11a*-3’-UTR constructs (200 ng) along with *miR-218–2-3p* using Lipofectamine 2000. The Dual-luciferase reporter assay system (Promega, Madison, WI) was used to quantify the luminescent signal using a luminometer (Glomax; Promega). Each value from the firefly luciferase assay was normalized to the Renilla luciferase assay value from the co-transfected phRL-null vector (Promega).

### Statistical analysis

All quantitative results were obtained from at least three independent experiments and these data are expressed as mean with standard deviation values. One-way ANOVA, non-parametric Kruskal–Wallis, or Student’s t-test was performed to assess statistical significance. A Tukey post-test or Dunn’s post-test was employed using GraphPad Prism 8.02 (La Jolla, CA). Student’s t-test was performed for two-group comparisons and one-way ANOVA, or Kruskal–Wallis test was used for multiple comparisons with the Tukey post-test for ANOVA and Dunn’s post-test for Kruskal–Wallis. *P* values of < 0.05 were considered statistically significant. *P* values of < 0.05, < 0.005, and < 0.0001 are denoted by *, **, and ***, respectively.

## Results

### SLIT3 induced by TLR4 activation in OCPs, which underlies the TLR4-mediated regulation of OC commitment

**Fig. 1:**
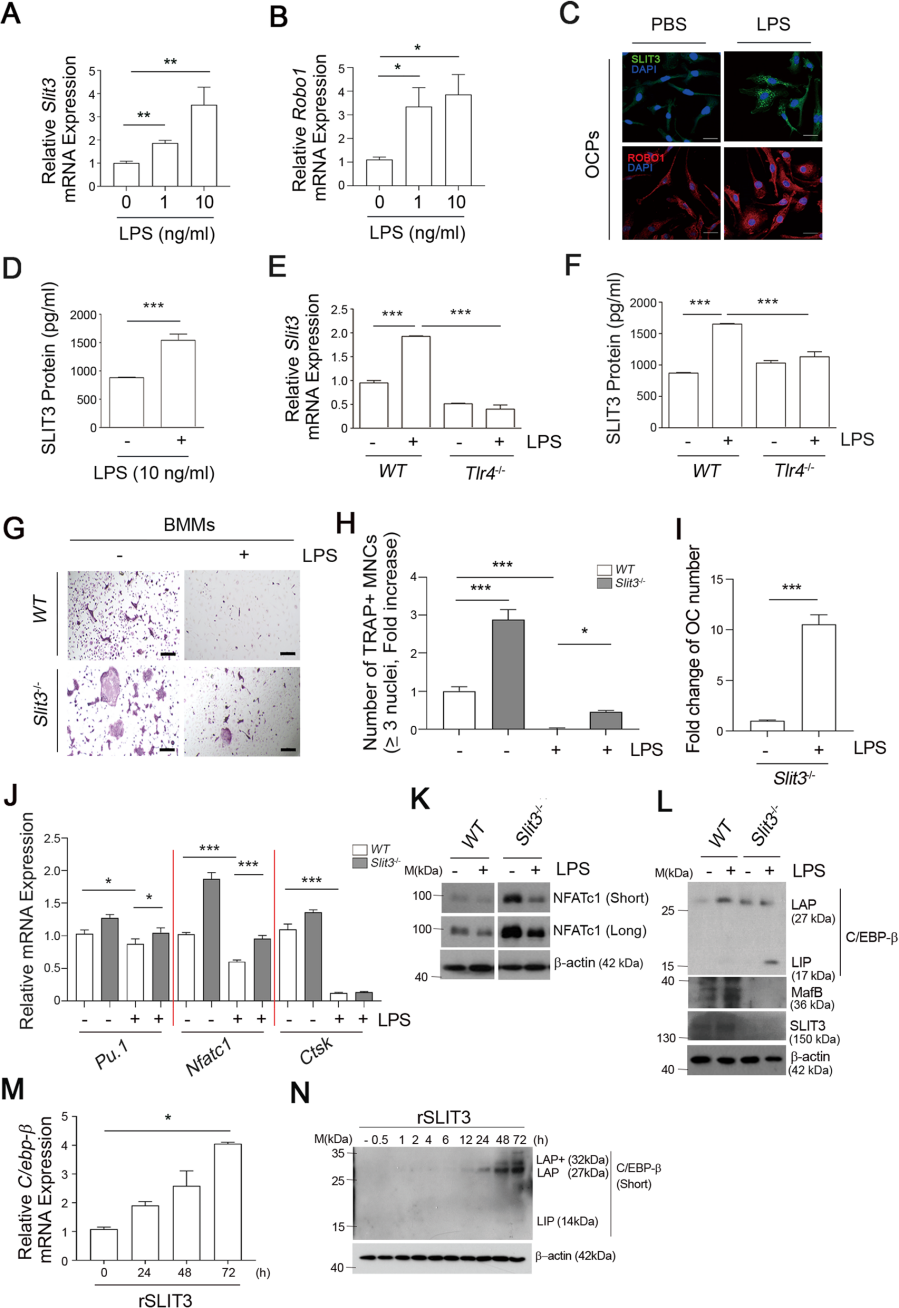
Enhanced SLIT3-ROBO1 signaling induced by LPS in OCPs regulates osteoclast differentiation by switching the C/EBP-β isoform. **A, B** OCPs were incubated with LPS for 4 h and the *Slit3* (**A**) and *Robo1* (**B**) mRNA levels were determined by qPCR. **C** OCPs were incubated with PBS or 10 ng/mL LPS for 6 h and immunofluorescence analysis was performed to detect SLIT3 and ROBO1. Scale bar, 20 μm. **D** OCPs were incubated with or without LPS for 24 h and the SLIT3 level was measured by ELISA. **E, F**. LPS-dependent SLIT3 upregulation was analyzed by qPCR (**E**) and ELISA (**F**) in OCPs isolated from *WT* or *Tlr4*^−/−^ mice in the presence of 10 ng/mL LPS. **G-I** OCPs isolated from *WT* or *Slit3*^−/−^ mice were cultured in a medium containing RANKL and LPS. After TRAP staining (**G**), the number of TRAP^+^ MNCs in the panel was quantified (**H**) and the increase of OC numbers in *Slit3*^−/−^ (*Slit3*^−/−^/*WT*) was calculated with LPS stimulation or not (**I**). Scale bar, 200 μm. **J** The mRNA expression of *Pu.1*, *Nfatc1*, and *Ctsk* were measured in pOCs from *WT* or *Slit3*^−/−^ mice, which had been incubated with or without LPS in a medium including M-CSF and RANKL for 24 h. **K** The NFATc1 protein level in cell lysates of *WT* or *Slit3*^−/−^ mature OCs, treated with or without LPS, was determined by immunoblotting. **L** The C/EBP- β protein isoforms LAP, LIP, and MafB, in addition to SLIT3 and β-actin, were analyzed. **M, N** OCPs were cultured with M-CSF in the presence of rSLIT3. The mRNA level of *C/ebp-β* was determined by qPCR (**M**), and the protein level of the LAP, LIP isoforms, and actin were analyzed by immunoblotting (**N**). Data are shown as mean ± s.d., * *P* < 0.05, **P < 0.005, ***P < 0.0001. All representative data from three independent experiments are shown

To better define the mechanism of regulation by TLR4 in the OC differentiation program, the factors that emerged from TLR4-activated OCPs were of considerable interest. The expressions of *Slit3* and *Robo1*, a receptor for SLIT3, were found to be markedly increased in OCPs by TLR4 activation with LPS at the mRNA level (Figs. 1A and B). On immunocytochemistry analysis, LPS treatment increased SLIT3 and ROBO1 protein levels in the mouse OCPs (Fig. 1C). We also observed an enhanced secretion of SLIT3 protein following LPS stimulation, as analyzed by ELISA (Fig. 1D) and that this LPS-mediated SLIT3 induction was absent in *Tlr4*-deficient OCPs isolated from *Tlr4*-deficient (*Tlr4*^*−/−*^) mice both at the mRNA (Fig. 1E) and protein levels (Fig. 1F). The prominent increase in SLIT3 by LPS exposure in OCPs suggested a potent role of this protein in TLR4-mediated OC regulation. We observed the role of SLIT3 during OC differentiation under LPS stimulation by using OCPs obtained from *Slit3*^−/−^ mice, generated as described previously [26]. In the presence of LPS, RANKL-induced OC differentiation was dramatically diminished in WT OCPs as shown by a reduced number of TRAP staining-positive MNCs (Figs. 1G and H), indicating an inhibitory role of TLR4 in OC differentiation in accordance with the previous study [16]; however, this inhibition effect was significantly reduced in *Slit3* deficient OCPs (Figs. 1H and I). At that time, the mRNA expression levels of OC-specific genes including *Pu.1*, *Nfatc1*, and cathepsin K (*Ctsk*) in OCs derived from the *Slit3* deficient OCPs were higher than those in WT OCPs, in the presence of LPS (Fig. 1J). Accordingly, the level of NFATc1 protein, a representative osteoclastogenic master gene, was higher in OCs derived from *Slit3*^−/−^ OCPs than from *WT* OCPs (Fig. 1K). An increase in the LIP/LAP ratio induces transcription factors related to OC commitment and leads to OC differentiation [34]. Notably, LPS stimulation increased the protein level of the C/EBP-β isoform LAP (Fig. 1L), which limits the osteoclastic potential of RANKL [35]. However, the *Slit3* deficient OCPs did not induce an increase in the protein level of the C/EBP-β isoform LAP compared to *WT* in the presence of LPS, but increased the LIP isoform, which is responsible for OC differentiation under RANKL stimulation (Figs. 1L). Also, we silenced the *Slit3* gene in OCPs by siRNA and differentiated them into OCs by culturing with M-CSF and RANKL in the presence or absence of LPS for four days. We confirmed siRNA knockdown of *Slit3* in LPS-treated cells and verified the knockdown by qPCR and SLIT3 ELISA assay (Figure S1A, B). *Slit3*-knockdown OCPs showed an increased number of TRAP^+^ MNCs relative to the OCs differentiated from control siRNA-transfected OCPs in response to RANKL in the presence or absence of LPS, and this increase was significantly enhanced with LPS stimulation (Figures S1C-E). *Slit3*-knockdown OCPs showed a higher increase in transcript levels of the OC marker genes, *Pu.1*, and *Nfatc1* during RANKL-induced OC differentiation, even in the presence of LPS, compared with the control cells (Figure S1F). Moreover, the protein expression of the C/EBP-β isoform LIP was increased in *Slit3*-knockdown OCPs upon LPS stimulation, but not in control OCPs with RANKL stimulation (Figure S1G, H). Conversely, recombinant SLIT3 (rSLIT3) treatment in OCPs elevated the mRNA level of *C/ebp-β* (Fig. 1M) and only induced an increase in the C/EBP-β isoform LAP protein (Fig. 1N), indicating the augmentation of the C/EBP-β isoform LAP to LIP ratio by SLIT3.

### SLIT3 deficiency increases the population of OCPs after LPS injection in vivo

**Fig. 2:**
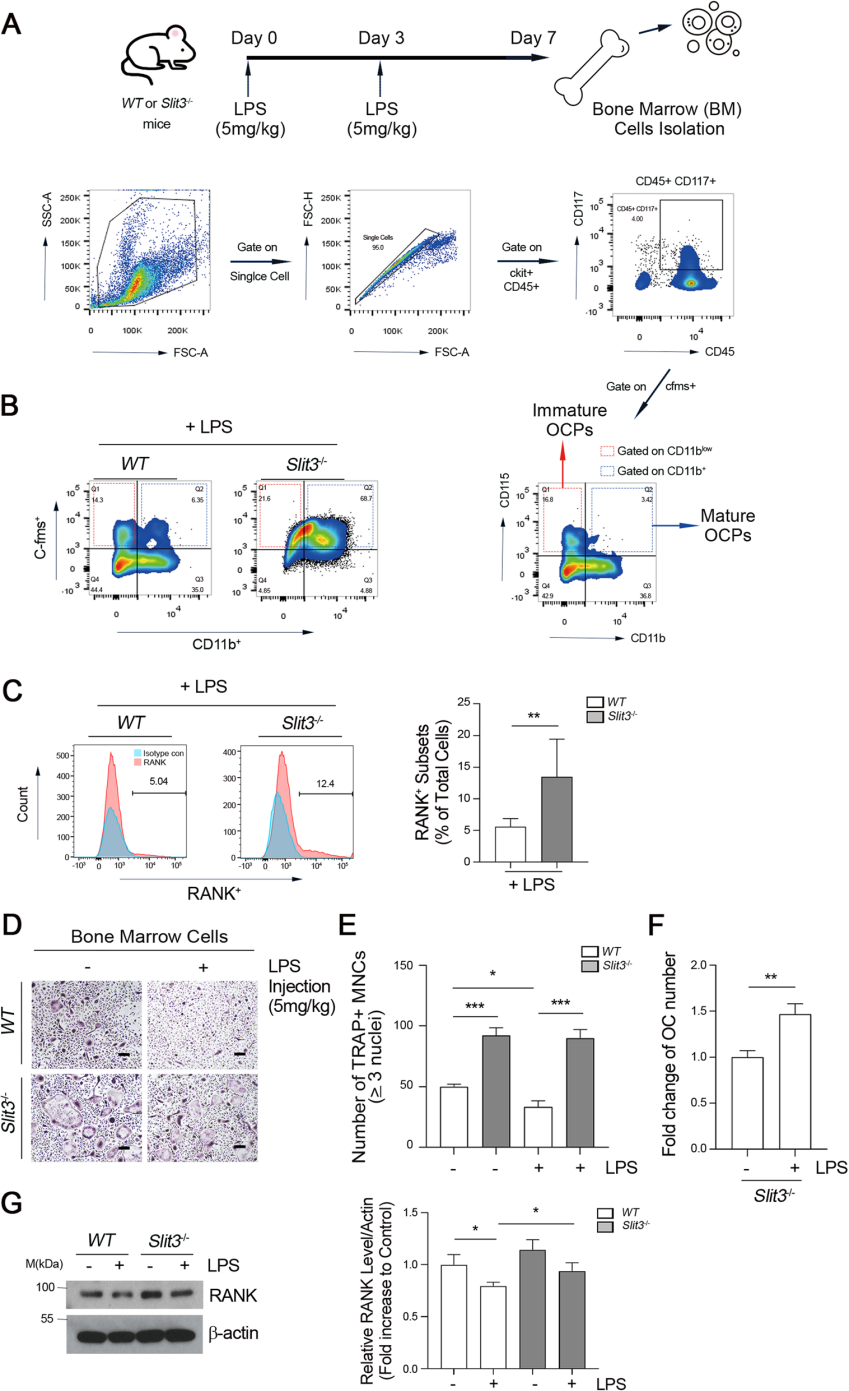
SLIT3 deficiency increases matured OCPs and upregulates RANK expression with LPS stimulation. **A-B** Bone marrow (BM) cells obtained from *WT* or *Slit3*^−/−^ mice that had received two LPS (5 mg/kg) intraperitoneal injections were analyzed by fluorescence-activated cell sorting (FACS) (**A**). Isolated BM cells were gated on ckit^+^CD45^+^, then cfms^+^CD11b^low^ and cfms^+^CD11b^hi^ populations were shown (**B**). **C** OCPs obtained from *WT* or *Slit3*^−/−^ mice were incubated with LPS (10 ng/ml) and cell surface RANK^+^ cell was observed by FACS analysis. **D-F** BM cells were incubated with M-CSF (30 ng/mL) and RANKL (10 ng/mL) for six days. Cells were then stained for TRAP (**D**) and the number of TRAP^+^ multinucleated cells (MNCs) containing more than three nuclei (**E**). The increase of OC numbers in *Slit3*^−/−^ (*Slit3*^−/−^ / *WT*) was calculated in PBS or LPS injection (**F**). Scale bar, 100 μm. **G** OCPs isolated from *WT* or *Slit3*^−/−^ mice were incubated with or without 10 ng/mL LPS and RANK expression was detected and quantified by immunoblotting against RANK- and β-actin-specific antibodies. Densitometry quantification of RANK compared to Actin is represented (*right*). Data are shown as mean ± s.d., *P < 0.05, **P < 0.005, ***P < 0.0001. All representative data from three independent experiments are shown

Given our observations that LPS-dependent induction of SLIT3 modulates the OC forming potential of OCPs by switching the C/EBP-β isoform, we reasoned that SLIT3 might affect the OC commitment status. The steps involved in the commitment of OCPs can be traced with surface markers for OCP maturation [36, 37] (i.e., ckit^+^cfms^+^CD11b^low^ cells for immature OCPs, and ckit^+^cfms^+^ CD11b^hi^ cells for mature OCPs) (Fig. 2A). In the present experiments, we observed that the frequency of mature OCPs was further increased in BM cells from *Slit3* knockout mice (*Slit3*^−/−^) mice compared to those from *WT* mice after an LPS challenge (Fig. 2B). The surface expression of RANK was higher in OCPs obtained from *Slit3*^−/−^ mice compared to *WT* OCPs under LPS stimulation. In addition, the population of RANK^+^ OCPs was increased in the *Slit3*^−/−^ mice (Fig. 2C). Moreover, we differentiated BM cells isolated from PBS or LPS-challenged *WT* or *Slit3*^−/−^ mice into OCs by culturing with M-CSF and RANKL for six days (Figs. 2D-F). RANKL-induced OC differentiation was increased in the BM cells from *Slit3*^−/−^ mice as shown by an increased number of TRAP staining-positive MNCs (Figs. 2D and E) and this effect was increased with LPS injection (Fig. 2F). This suggested that a SLIT3 deficient inflammation status leads to an enhanced osteoclastogenic potential, accompanied by an increase in the OCP population in vivo. Previous studies have indicated that the M-CSF signaling induces RANK expression in macrophages leading to OC commitment [2]. It was reported that LPS reduces the expression of RANK (*Tnfrsf11a*) in BMMs and regulates further OC differentiation [38]. Consistently, we herein confirmed a reduction in the protein expression level of RANK (Fig. 2F) in BMMs upon LPS stimulation. However, *Slit3*^−/−^ BMMs expressed a much higher level of RANK protein upon LPS stimulation than WT OCPs (Fig. 2G). These results suggest that the SLIT3 induced by LPS attenuates RANK expression and thereby dampens OC commitment.

### Concomitant *miR-218–2-3p* directly suppresses RANK expression by targeting its 3’-UTR

**Fig. 3:**
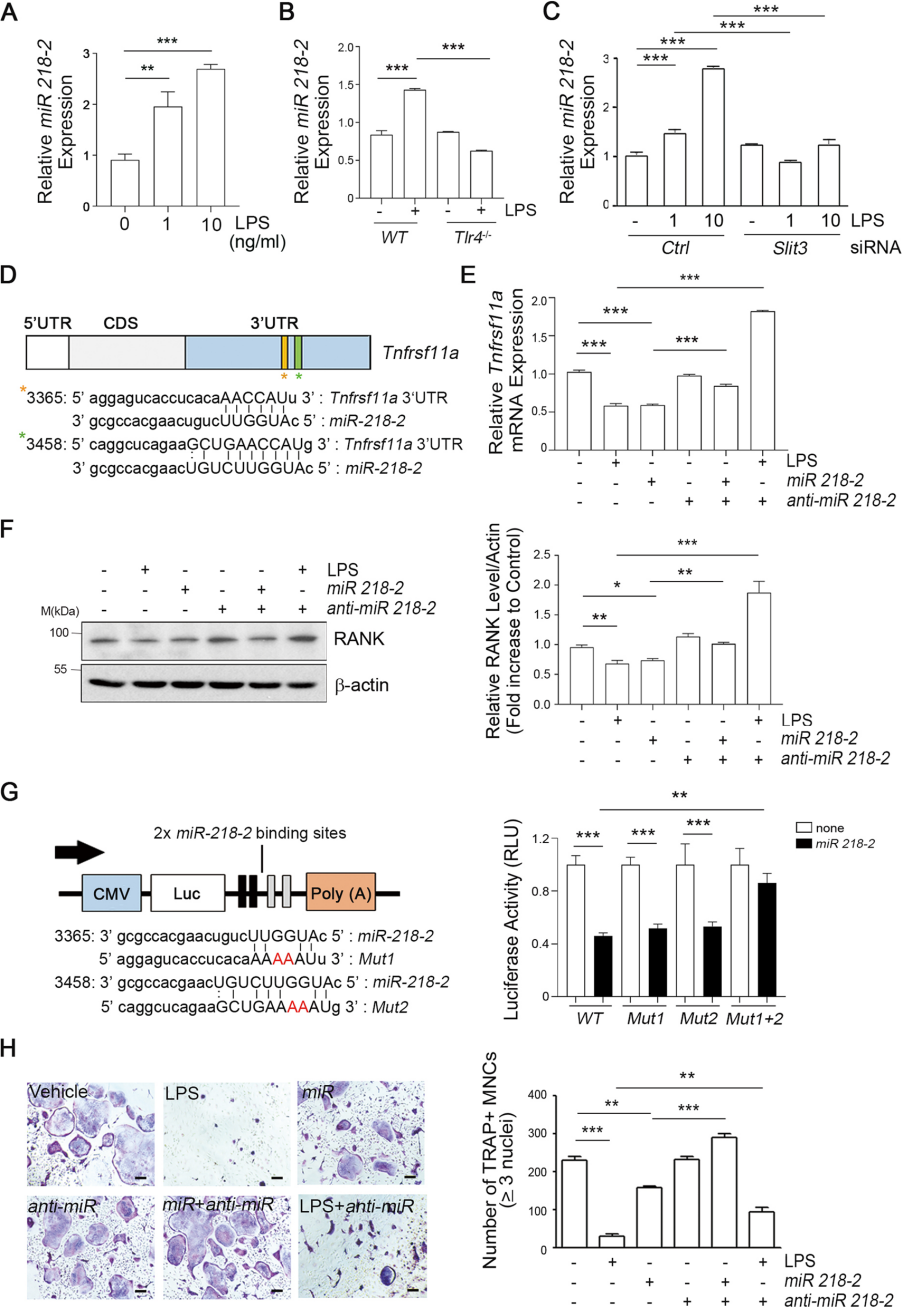
TLR4 activation enhances *miRNA-218* in OCPs, which suppresses osteoclast differentiation by directly inhibiting RANK expression. **A** The mouse OCPs were incubated with LPS for 4 h and *miR-218–2* expression was measured by qPCR. **B** *WT* or *Tlr4*^−/−^ OCPs were incubated with 10 ng/mL LPS for 4 h and *miR-218–2* expression was determined using qPCR. **C** *WT* or *Slit3* knockdown OCPs were incubated with 0, 1, or 10 ng/mL LPS for 4 h and *miR-218–2* expression was then assayed. **D** Schematic representation of *miR-218–2-3p* targeting sites in the *Tnfrsf11a* 3’-UTR. **E, F** OCPs were transfected with or without the *miR-218–2-3p* mimic and *miR-218–2-3p* inhibitor (100 nM), and then incubated with or without 10 ng/mL LPS for 4 h and subjected to qPCR analysis of the relative *Tnfrsf11a* mRNA expression levels (**E**). Moreover, these transfected cells were incubated with or without LPS for 24 h and then the RANK protein expression level was analyzed by immunoblotting and quantified with the ImageJ software (**F**). **G** Schematic representation of firefly luciferase constructs containing the CMV promoter, luciferase coding region, and a fragment of the *Tnfrsf11a* 3’-UTR (**G, left**). NIH3T3 cells were co-transfected with *miR-218–2-3p*, firefly luciferase constructs (*Tnfrsf11a* 3’-UTR WT, Mut1, Mut2, or Mut1 + 2), and Renilla luciferase control for the dual-luciferase assay. The relative luciferase activity represents the dual luciferase activity ratio (i.e., firefly/Renilla luciferase). WT, wild type; Mut 1, mutation at site 1; Mut 2, mutation at site 2; Mut1 + 2, mutation at both sites 1 and 2 (**G, right**). **H** The transfected OCPs with or without *miR-218–2-3p* mimic or *miR-218–2-3p* inhibitor were cultured with M-CSF and RANKL in the presence or absence of LPS (10 ng/mL), and in the presence or absence of *anti-miR* (Qiagen). These cells were then fixed, stained for TRAP (**H, left**), and the number of TRAP^+^ MNCs containing three or more nuclei (MNCs; ≥ 3 nuclei) were counted under a light microscope (**H, right**). Scale bar, 100 μm. Data are shown as mean with s.d., *P < 0.05, **P < 0.005, ***P < 0.0001. All representative data from three independent experiments are shown

It was reported that SLIT3 encodes an intronic miRNA, *miR-218–2* [39]. In the current analysis, LPS was found to dramatically increase the expression of *miR-218–2-3p* (Fig. 3A) in OCPs and was not detectable in *Tlr4*-deficient OCPs (Fig. 3B). Furthermore, the expression of *miR-218–2-3p* was decreased by the *Slit3*-knockdown (Fig. 3C). To define the putative targets of *miR-218–2*, we conducted a search using the TargetScan [40] and miRANDA [41] algorithms. Among the predicted targets, RANK was found to harbor two potential binding sites for *miR-218–2* in its 3’-UTR (Fig. 3D, denoted by the yellow and green stars). We subsequently determined whether *miR-218–2* affects RANK expression by transfecting OCPs with a mimic or inhibitor of this miRNA. In the presence or absence of LPS, the mRNA (Fig. 3E) and protein levels (Fig. 3F) of RANK were significantly suppressed by *miR-218–2* overexpression, respectively. Importantly, the *miR-218–2*-mediated reduction in RANK expression was completely abrogated by the transfection of mouse OCPs with an *anti-miR-218–2*. To further confirm the direct interaction between *miR-218–2* and the *Tnfrsf11a*, we introduced the predicted binding sites for *miR-218–2* (-3p) within the *Tnfrsf11a* 3’-UTR sequence into a pMIR-CMV-Luc reporter vector (Fig. 3G, *left*), transiently transfected this construct into murine NIH3T3 cells, and then conducted a luciferase reporter assay (Fig. 3G, *right*). *miR-218–2* overexpression significantly inhibited the luciferase activity of reporter constructs containing *WT* 3’-UTR-*Tnfrsf11a* (Fig. 3G, *right*). To verify the suppressive effect of miR-218–2 on the RANK 3’-UTR, we constructed three mutant subtypes of this region (*Tnfrsf11a* Mut1, Mut2, or Mut1 + 2) (Fig. 3G, *right*). The mutation of either binding site alone (Mut1 or Mut2) did not effectively block the suppressive effects of *miR-218–2* on the luciferase activity driven by the *Tnfrsf11a* 3’-UTR. However, when both *miR-218–2* binding sites (Mut1 + 2) in the potential target UTRs were mutated, this suppressive effect was abolished, indicating that *miR-218–2* regulates *Tnfrsf11a* via the two predicted target sites in its 3’-UTR (Fig. 3G, *right*). Furthermore, we observed that *miR-218–2* overexpression markedly suppressed OC formation, and that this was restored by the overexpression of *anti-miR-218–2*. The *anti-miR-218–2* molecule also rescued the inhibitory effects of LPS on OC formation (Fig. 3H). These data suggest that SLIT3 induction by TLR4 activation is aligned with the expression of *miR-218–2* to target *Tnfrsf11a* directly and that this further suppresses OC commitment pathways.

### A SLIT3 deficiency enhances OC formation upon LPS stimulation in vitro and LPS-induced bone loss in vivo

**Fig. 4:**
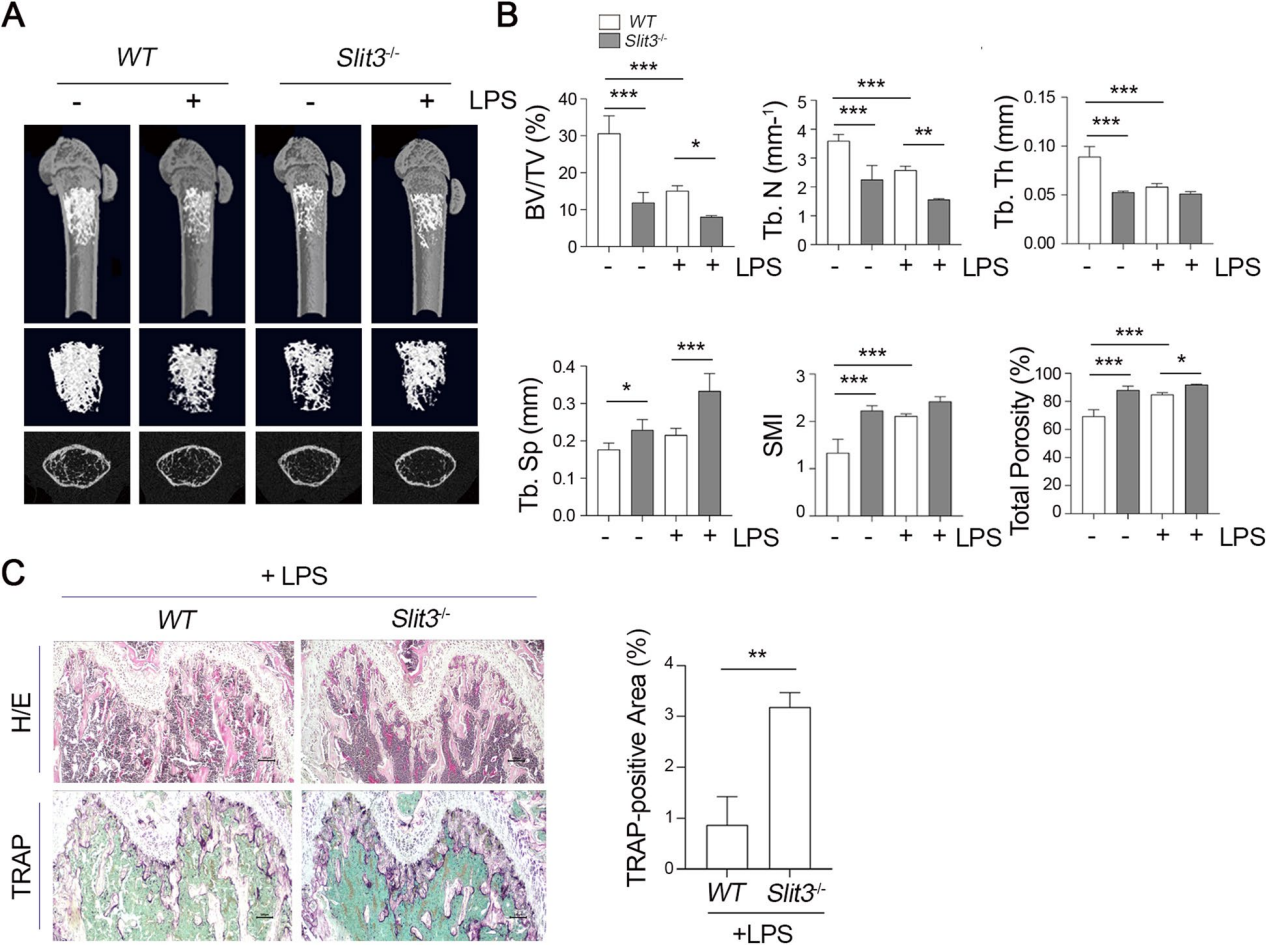
SLIT3 deficiency is associated with increased osteoclastic potential under LPS stimulation. **A, B** *WT* and *Slit3*^−/−^ mice at 8 weeks of age were injected intraperitoneally with PBS or LPS. Both hind limbs were examined by micro-CT imaging. Representative images of the trabecular bone of femurs from each group are presented (*n* = 4 ~ 6 images taken in total, one image from each of 4 ~ 6 mice). Histograms representing the three-dimensional structural parameters of the femurs in a micro-CT reconstruction of the metaphyses of distal femurs (**A**). (**B**) Three-dimensional morphometric analysis of bone parameters including bone volume per tissue volume (BV/TV), trabecular number (Tb. N), trabecular thickness (Tb. Th), trabecular separation (Tb. Sp), structure model index (SMI), and total porosity (%) were calculated from femur sections using the micro-CT analysis program. The results are presented as the means ± s.d. of 4 to 6 male mice/group. **C** Hematoxylin and eosin (H/E) and TRAP (purple) staining of hind limb sections from *WT* and *Slit3*^−/−^ mice. Scale bar, 100 μm. Data are shown as mean with s.d., *P < 0.05, **P < 0.005, ***P < 0.0001. All representative data from three independent experiments are shown

We finally confirmed the role of SLIT3 in TLR4-mediated OC differentiation using *Slit3*^−/−^ mice. Distinct from the in vitro inhibitory effect of LPS during OC commitment, LPS challenge enhances bone loss in vivo due to the presence of osteoclastogenic factors in the inflammatory bone microenvironment [10]. Based on our current findings of the increase in OCP population in *Slit3*-deficient mice (Fig. 2), we reasoned that SLIT3 deficiency might deteriorate LPS-induced inflammatory bone loss in vivo. Indeed, following LPS injection, *Slit3*^−/−^ mice displayed a much smaller femoral bone volume and reduced bone mass compared with *WT* mice (Fig. 4A). We also observed a significantly reduced BV/TV and trabecular number (Tb. N) in *Slit3*^−/−^ mice than those in the *WT* injected with LPS (Fig. 4B, *top*). Bone resorptive parameters such as total porosity, and the ratio of trabecular separation/spacing (Tb. Sp) in *Slit3*^−/−^ mice were consequently increased compared to *WT* with LPS injection (Fig. 4B, *bottom*) with an increase in TRAP staining-positive OCs in vivo in trabecular bone (Fig. 4C). Taken together, our findings indicate that a SLIT3 deficiency leads to bone loss along with increased OC formation in vivo, implying a protective role for this protein in the bone erosion elicited by TLR4 activation.

## Discussion

Like many chronic inflammatory states in the bone microenvironment [42], TLR4 activation plays a crucial role in producing various pro-inflammatory cytokines such as TNF-α, IL-6, and IL-1β in macrophages, which stimulate OC formation and contribute to the pathology of inflammatory bone diseases [7, 11, 12, 43]. Given that macrophages are bipotential precursors that can form either inflammatory macrophages or bone-resorbing OCs under normal physiological and disease conditions [44–46], the monocyte-to-macrophage transition, in close communication with inflammation processes elicited by TLR4 activation, can serve as a cue for OC formation in the presence of RANKL [12, 43]. During the innate immune responses to microbial infection, TLR4 mediates highly regulated inflammatory responses by limiting the extent of inflammation to avoid excessive toxicity that could lead to tissue damage [47]. The association of single nucleotide polymorphisms in the coding region of the *Tlr4* gene with osteoporosis [48] and chronic periodontitis [49] supports the notion of a protective role of TLR4 in bone metabolism. Similarly, it is plausible that mechanisms for the maintenance of normal bone immunity to prevent undesirable bone damage by TLR4 activation exist, but direct evidence for this is still lacking.

**Fig. 5:**
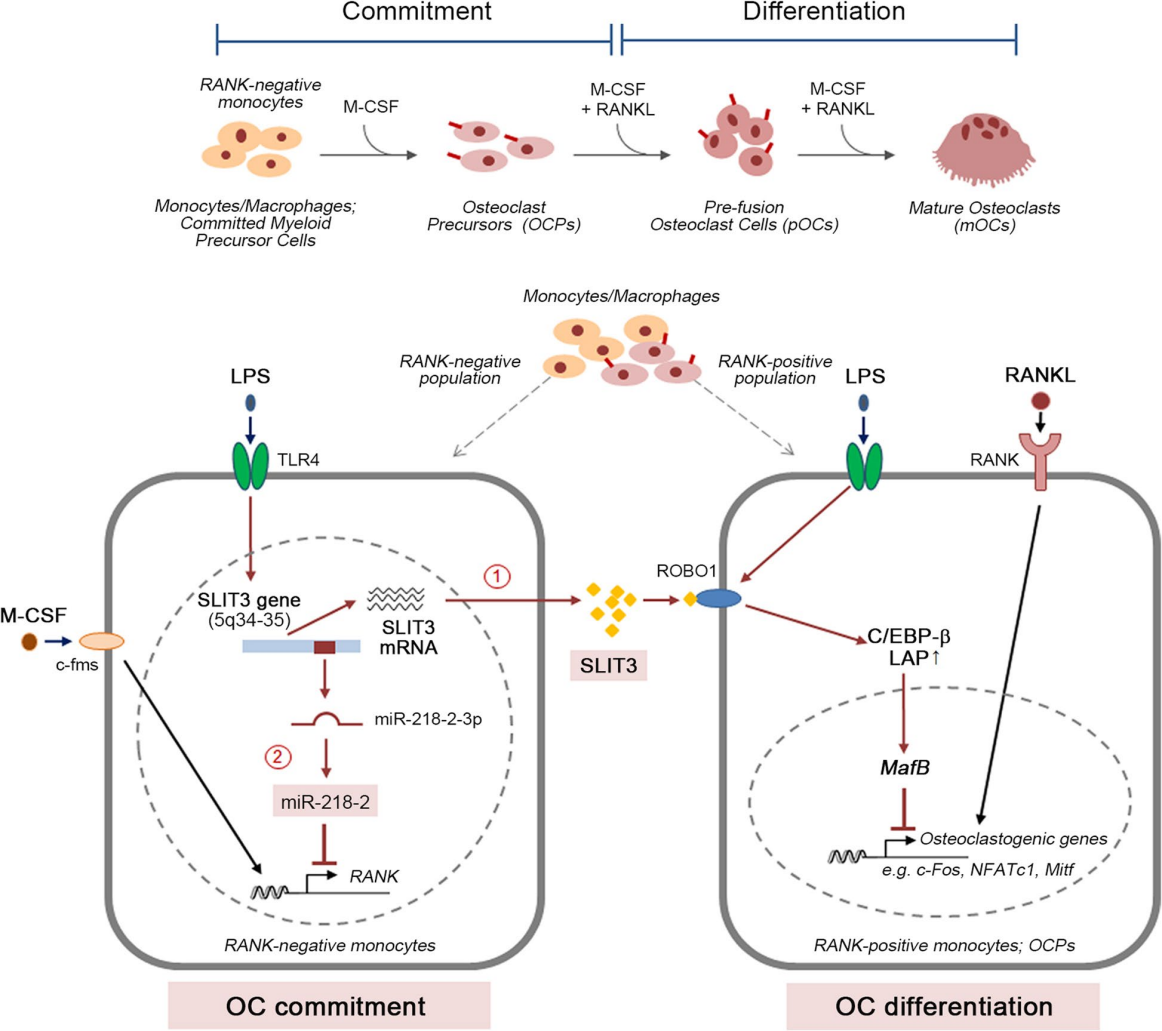
Schematic diagram of SLIT3 function upon LPS stimulation of OCPs. Committed myeloid precursor cells (monocytes/macrophages) with RANK-negative monocytes undergo commitment into RANK-positive monocytes, which is an osteoclast precursor cells (OCPs) in M-CSF response and differentiation into mononuclear pre-fusion osteoclast cells (pOCs) and multinucleated OCs (mOCs) is further modulated by RANKL and M-CSF signaling. However, LPS stimulation through TLR4 upregulates SLIT3/ROBO1 expression in OCPs, and this SLIT3-ROBO1 signaling raises the ratio of the C/EBP-β isoform LAP to LIP protein level, and induces MafB expression, leading to restrained osteoclast differentiation potential in RANKL-primed OCPs. Furthermore, SLIT3 induction concomitantly increases *miR-218–2-3p* expression, which directly inhibits the expression of RANK, and synergistically controls osteoclastic potential in OCPs

Although TLR4 activation stimulates the differentiation of monocytes into macrophages, it displays a distinctive function in macrophages during the OC commitment phase such as the inhibition of OC commitment from macrophages or the enforcement of OC differentiation from RANKL-primed OCPs [11]. Based on our current findings and the reported evidence to date, we propose a molecular mechanism by which TLR4 activation hinders the commitment phase of monocytes/macrophages to OCP formation and further OC differentiation (Fig. 5). In monocytes/macrophages, TLR4 activation triggers the concomitant expression of *Slit3* and *miR-218–2* (especially 3p), as well as *Robo1*. First, *miR-218–2* suppresses the expression of RANK, a critical factor for OC commitment (pathway 2). Second, secreted SLIT3 proteins bind to ROBO1, a factor that can affect RANKL-induced signaling, via the switching of C/EBP-β isoforms, which is a known process in OC differentiation [34, 50]. This SLIT3-ROBO1 axis increases the ratio of LAP to attenuate the RANKL-induced expression of the OC master gene, *Nfatc1*, via MafB activation [51] and thereby restrains OC differentiation (pathway 1). However, in the inflammatory bone environment, the protective function of the SLIT3-ROBO1 axis may not be effective in suppressing OC differentiation via pathway 1 due to the crosstalk between osteoblasts (OBs) and OCs on the commitment and differentiation of OCs [11]. TLR4 activation in OB enhances the expression of M-CSF and RANKL to promote the conversion of RANK-expressing OCPs into OCs [11, 14]. Additionally, TLR4 activation directly reinforces RANKL-mediated intracellular signaling by activating the ROS/PP2A/NF-κB axis in OCPs [52]. In this scenario, the effect of the SLIT3-ROBO1 axis signaling in skewing the fate of OCPs can be masked. Nevertheless, our series of evidence support the notion that SLIT3 deficiency affects the OC commitment phase and contributes to excessive bone damage.

During normal inflammatory processes, activated macrophages can adopt two distinct phenotypes, i.e., classical pro-inflammatory M1 macrophages and alternative anti-inflammatory M2 macrophages [53]. The M1 to M2 macrophage transition is a tightly regulated process essential for the resolution of inflammation and wound healing [54]. Hence, an imbalance in macrophage M1-M2 polarization can affect a range of inflammatory disease conditions. Although both activated polarized M1 and M2 macrophages act as OCPs [55], the pro-inflammatory cytokine TNF-α, operating in chronic inflammatory bone diseases such as rheumatoid arthritis, can switch the differentiation of M2 to M1 macrophages to expand the OCP pool, thereby enhancing the OC forming potential [1]. The identification of SLIT3 as a cytokine secreted from M2-like macrophages in adipose tissue [56] implies that LPS-stimulated macrophages may have an M2-like phenotype with weak OC forming potential rather than M1.

Recently, the SNP rs10036727 in the *SLIT3* gene was reported to be associated with osteoporosis at the femoral neck in postmenopausal women [57], supporting the notion of a protective role of SLIT3 in bone metabolism. More importantly, there is reliable evidence that SLIT3 dysfunction may skew the balance in bone metabolism during bone disease progression. In the present study, *Slit3*-deficient mice showed enhanced bone loss and were more sensitive to LPS-induced bone loss in vivo than their *WT* counterparts (Figs. 4A and B). Mechanistically, this outcome may be caused by SLIT3 and ROBO1 induction in response to LPS (Fig. 1). We found that the SLIT3-ROBO1 signaling axis increased the LAP and not the LIP isoform of C/EBP-β (Fig. 1N) and, conversely, that a deficiency of *Slit3* in OCPs led to a prominent increase in the LIP level in response to LPS, forcing the formation of OCs even in the presence of LPS, as evidenced by the increased levels of OC-related genes (Fig. 1J). The ratio of the LAP to LIP C/EBP-β isoforms is reportedly associated with the OC differentiation program [34]. The elevation of the LIP isoform of C/EBP-β inactivates MafB expression to induce osteoclastic transcription factors including c-Fos, MITF, and NFATc1, and this process is known to be controlled by the mTOR pathway [34]. The inhibition of mTOR by rapamycin decreases the translation of the C/EBP-β LIP isoform [58, 59] and thus decreases OC formation [34]. In mouse embryonic fibroblasts and HeLa cells, Rac1 has also been reported to bind directly to mTOR and simultaneously regulate mTORC1 and mTORC2 activity by colocalizing with these proteins at specific membranes [60]. Since the SLIT3-ROBO1 signaling pathways decrease Rac1 activity in OCs [26, 61], it can be reliably concluded that SLIT3 can affect the mTOR signaling associated with reduced Rac1 activity in OCPs. However, further investigations are needed to validate this. Taken together, our current findings and prior evidence indicate that there is a TLR4-dependent elevation of the SLIT3-ROBO1 axis in OCPs, which in turn may affect OC differentiation via the switching of C/EBP-β isoforms.

It is well known that host gene functions are commonly regulated by small noncoding RNAs, the miRNAs, to inhibit translation or promote mRNA degradation by binding to the 3′-UTRs of their target mRNAs, resulting in a “fine-tuning” of gene expression [62]. Several lines of evidence also suggest that miRNAs participate in OC differentiation and bone resorption [63, 64]. For example, *miR-21* is highly expressed in OCPs and simultaneously promotes osteoclastogenesis and osteoclastic apoptosis by targeting programmed cell death protein 4 [64]. *miR-223* modulates OC differentiation by regulating a transcriptional repressor, nuclear factor I-A, and the expression of the M-CSF receptor, c-fms [65]. Moreover, both *miR-155* and *miR-503* inhibit OC differentiation by targeting PU.1, MITF [66], or RANK [67]. Recently, Qu et al*.* reported that *miR-218* negatively regulates osteoclastogenesis through the partial suppression of p38-MAPK-c-Fos-NFATc1 signaling [68]. *MiR-218* is also found to be downregulated in CD14^+^ peripheral blood mononuclear cells from patients with postmenopausal osteoporosis [68], further emphasizing its role in bone metabolism. Mature *miR-218* effectors include *miR-218–1* and *miR-218–2*, which are encoded within the introns of *Slit2* and Slit3, respectively [69]. In the current study, *miR-218–2* and SLIT3 were found to be induced by TLR4 activation in OCPs (Figs. 3A and B) and then suppress M-CSF-dependent RANK expression by binding to two target sites in the *Tnfrsf11a*-3’-UTR (Fig. 3D). This in turn resulted in a decreased OC formation (Fig. 3H). Furthermore, the *Slit3*-knockdown in OCPs inhibited LPS-induced *miR-218–2* expression (Fig. 3C), indicating that *miR-218–2* expression is dependent on SLIT3 induction. Notably however, *miR-218–2* did not affect the expression of the inflammatory genes underlying LPS-stimulated biological functions (data not shown). A recent report has suggested that *Slit3* and *miRNA-218-2* are new candidate genes related to bone density [70], further indicating a likely functional association between intronic miRNAs and their host genes in bone metabolism. Hence, our present findings provide a mechanistic explanation for the regulatory role of TLR4 activation in OC commitment and new insights into the OC differentiation program and bone metabolism.

## Conclusions

TLR4 activation in OCPs induced the concomitant expression of SLIT3 and *miR-218–2* as well as SLIT3 receptor, ROBO1, resulting in the inhibition of osteoclast commitment. The expression of *miR-218–2* targets *Tnfrsf11a* (RANK) directly to suppress osteoclast commitment. Secreted SLIT3 decreased the RANKL-induced osteoclast commitment by switching C/EBP-β isoforms. *Slit3*-deficient mice displayed augmentation of osteoclast precursor populations in response to TLR4 activation, accompanied by the induction of osteoclast formation, implying the protective role of SLIT3-*miR-218–2* in bone erosion with TLR4 activation. Based on these findings, we suggest a mechanistic explanation for the role of SLIT3 controlling the TLR4-induced OC differentiation program.

### Supplementary Information

**Additional file 1:** **Fig. S1. **Depletion of *Slit3* increases the osteoclastic potential in OCPs by switching the C/EBP-β isoform. **A**, **B** OCPs were transfected with *control* siRNA or *Slit3*-targeting siRNA. After 24 h, transfected cells were incubated with or without LPS (10 ng/ml) and then assessed by qPCR (**A**) and ELISA assay (**B**) for siRNA-mediated downregulation of SLIT3. **C-E** Transfected cells by specific siRNA were incubated with M-CSF and RANKL in the presence or absence of LPS (10 ng/mL). Cells were then stained for TRAP (**C**) and the number of TRAP^+^ MNCs containing more than three nuclei (**D**). The cells were counted under a light microscope and the increase of OC numbers with *Slit3* deletion (*siSLIT3* / *siCtrl*) was calculated with LPS stimulation or not (**E**). Scale bar, 100 μm. **F-H** OCPs were incubated with M-CSF (30 ng/mL) alone for 24 h, transfected with *control* siRNA or *Slit3* siRNA for 24 h, and further incubated with RANKL (10 ng/mL) and LPS for 24 h. The transcript levels of *Pu.1*, *Nfatc1*, and *Ctsk* were then assayed by qPCR (**F**) and the protein levels of C/EBP-β, LIP, LAP, and β-actin were determined by immunoblotting (**G**) Densitometry quantification of LIP compared to LAP is represented (**H**). Data are shown as mean ± s.d., * *P* < 0.05, **P < 0.005, ***P < 0.0001. All representative data from three independent experiments are shown.

## Data Availability

The datasets used and/or analyzed during the current study are available from the corresponding author on reasonable request.

## Abbreviation

TLR4: Toll-like receptor 4
OCP: Osteoclast precursor
pOC: Pre-fusion osteoclast cell
mOC: Mature osteoclast
pOB: Pre-osteoblast
LPS: Lipopolysaccharide
OC: Osteoclast
BMM: Bone marrow-derived macrophage
TRAP^+^ MNC: TRAP-positive multinuclear cell
M-CSF: Macrophage colony-stimulating factor
RANK: Receptor activator of nuclear factor κB
RANKL: Receptor activator of nuclear factor κB ligand
ROBO: Roundabout
*Tlr4*^−^^/^^−^: *Tlr4* Knockout
rSLIT3: Recombinant SLIT3
*Slit3*^−^^/^^−^: *Slit3* Knockout
BV/TV: Bone volume per tissue volume
Tb. N.: Trabecular number
Tb. Th.: Trabecular thickness
Tb. Sp.: Trabecular separation
SMI: Structure model index
OB: Osteoblast
WT: Wild-type
BM: Bone marrow
FBS: Fetal bovine serum
qPCR: Quantitative real-time PCR
PBS: Phosphate buffered saline
ELISA: Enzyme-linked immunosorbent assay
PFA: Paraformaldehyde
CT: Micro-computed tomography
MNC: Multinucleated cell
FACS: Fluorescence-activated cell sorting
H/E: Hematoxylin and eosin
