# Supplementary material for: Concomitant induction of SLIT3 and microRNA-218–2 in macrophages by toll-like receptor 4 activation limits osteoclast commitment
Source: Cell Commun Signal. 2023 Aug 18;21:213. doi: 10.1186/s12964-023-01226-w (PMC10436635; doi:10.1186/s12964-023-01226-w)
Supplement: Supplementary file 2 — Additional file 1: Fig. S1. Depletion of Slit3 increases the osteoclastic potential in OCPs by switching the C/EBP-β isoform. A, B OCPs were transfected with control siRNA or Slit3-targeting siRNA. After 24 h, transfected cells were incubated with or without LPS (10 ng/ml) and then assessed by qPCR (A) and ELISA assay (B) for siRNA-mediated downregulation of SLIT3. C-E Transfected cells by specific siRNA were incubated with M-CSF and RANKL in the presence or absence of LPS (10 ng/mL). Cells were then stained for TRAP (C) and the number of TRAP+ MNCs containing more than three nuclei (D). The cells were counted under a light microscope and the increase of OC numbers with Slit3 deletion (siSLIT3 / siCtrl) was calculated with LPS stimulation or not (E). Scale bar, 100 μm. F-H OCPs were incubated with M-CSF (30 ng/mL) alone for 24 h, transfected with control siRNA or Slit3 siRNA for 24 h, and further incubated with RANKL (10 ng/mL) and LPS for 24 h. The transcript levels of Pu.1, Nfatc1, and Ctsk were then assayed by qPCR (F) and the protein levels of C/EBP-β, LIP, LAP, and β-actin were determined by immunoblotting (G) Densitometry quantification of LIP compared to LAP is represented (H). Data are shown as mean ± s.d., * P < 0.05, **P < 0.005, ***P < 0.0001. All representative data from three independent experiments are shown. [file 12964_2023_1226_MOESM1_ESM.docx]

## Supplementary Material

##
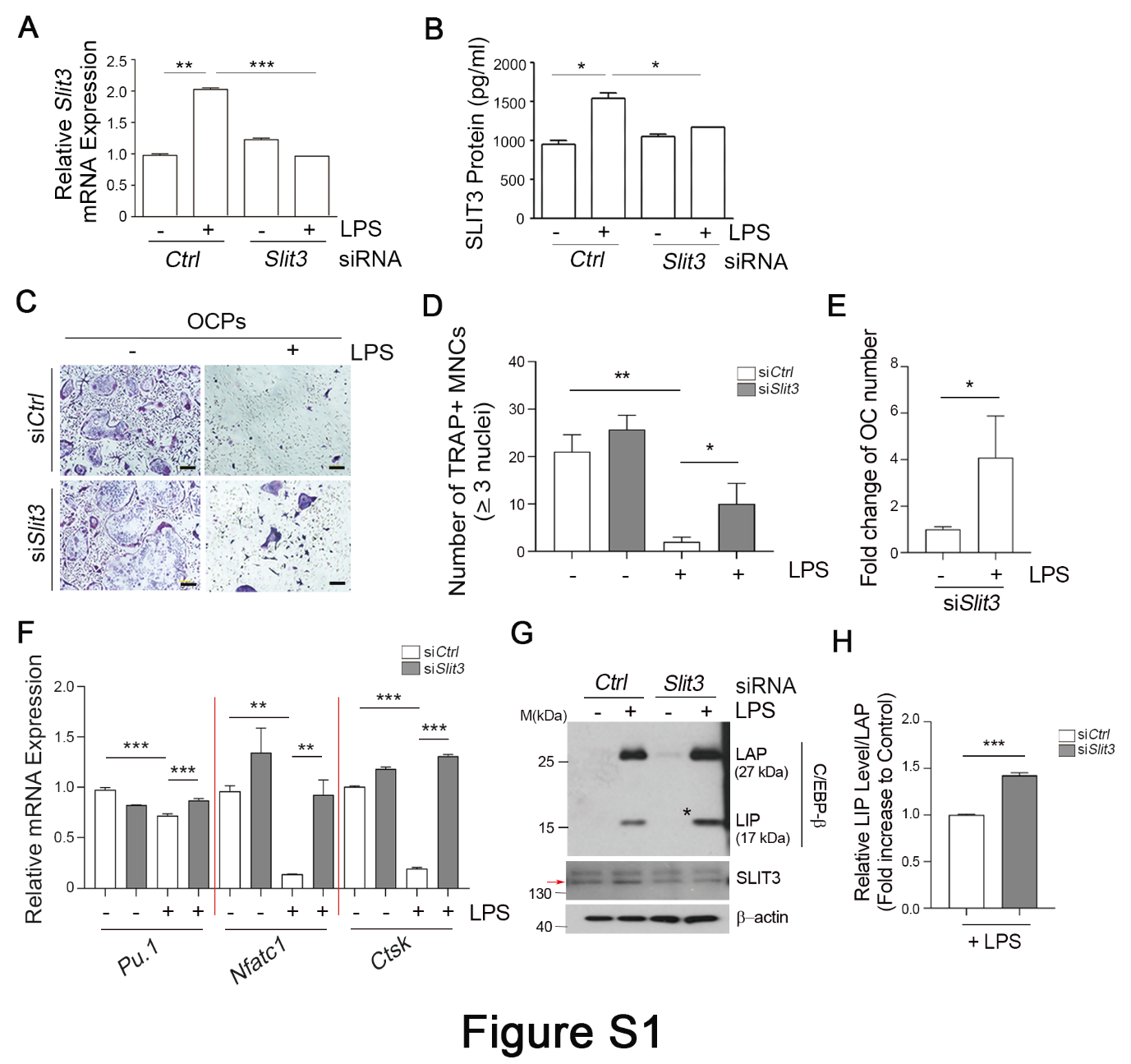


**Fig. S1.** Depletion of *Slit3* increases the osteoclastic potential in OCPs by switching the C/EBP-β isoform. **A, B** OCPs were transfected with *control* siRNA or *Slit3*-targeting siRNA. After 24 h, transfected cells were incubated with or without LPS (10 ng/ml) and then assessed by qPCR (**A**) and ELISA assay (**B**) for siRNA-mediated downregulation of SLIT3. **C-E** Transfected cells by specific siRNA were incubated with M-CSF and RANKL in the presence or absence of LPS (10 ng/mL). Cells were then stained for TRAP (**C**) and the number of TRAP^+^ MNCs containing more than three nuclei (**D**). The cells were counted under a light microscope and the increase of OC numbers with *Slit3* deletion (*siSLIT3* / *siCtrl*) was calculated with LPS stimulation or not (**E**). Scale bar, 100 μm. **F-H** OCPs were incubated with M-CSF (30 ng/mL) alone for 24 h, transfected with *control* siRNA or *Slit3* siRNA for 24 h, and further incubated with RANKL (10 ng/mL) and LPS for 24 h. The transcript levels of *Pu.1*, *Nfatc1*, and *Ctsk* were then assayed by qPCR (**F**) and the protein levels of C/EBP-β, LIP, LAP, and β-actin were determined by immunoblotting (**G**) Densitometry quantification of LIP compared to LAP is represented (**H**). Data are shown as mean ± s.d., * *P* < 0.05, ***P* < 0.005, ****P* < 0.0001. All representative data from three independent experiments are shown.
